# Evaluating the Impact of Early Career Academic Medicine Workshops on Medical Students’ Interest

**DOI:** 10.7759/cureus.41710

**Published:** 2023-07-11

**Authors:** Kelly Chang, Dhruvi Panchal, Jade Bowman, Shazia Sheikh, Hina Mohsin

**Affiliations:** 1 Medical Education, California University of Science and Medicine, Colton, USA

**Keywords:** mentorship program, interactive workshop, medical education research, medical education curriculum, graduate medical education (gme), academic medicine

## Abstract

Introduction

Academic medicine is an important field that has had a notable decline in physician interest. The aim of this study was to introduce academic medicine to medical students early in their careers with a workshop in the medical school setting, beyond conferences, to promote even greater interest in the field.

Methods

This workshop consisted of (1) an informational didactic session using a Microsoft PowerPoint presentation, (2) small-group breakout discussion sessions to review case scenarios, and (3) a faculty panel to provide personal anecdotes and advice to students. The authors administered online pre- and post-workshop surveys to the students. One workshop was presented to first-year medical students and another to second-year medical students at California University of Science and Medicine. Data were analyzed using the IBM SPSS Statistics 27.0 for Windows. Pre- and post-workshop survey question means were compared using a paired t-test.

Results

There were 104 pre-clerkship student attendees, 83 of whom were in their first year and 21 in their second. Within each class year, there was a statistical significance in pre- and post-workshop survey responses for questions one through four (p < 0.001, p < 0.001, p < 0.001, p < 0.001), but question five responses were not statistically significant (p = 0.78).

Conclusion

Academic medicine workshops held early in medical students' careers are an effective way to foster interest in the field. Implementing academic medicine scholars' programs, in addition to these workshops, can help provide guidance and resources for students who want to pursue a career in academic medicine.

## Introduction

Teaching and training are integral aspects of every physician's medical experience. It is not only essential to one's own education but also in treating future patients, collaborating with colleagues, and educating the next generation of physicians. Despite this importance, there is not enough formal training to adequately prepare physicians with these leadership skills [1]. Academic medicine is a field that focuses on improving patient care, mentorship, and developing research abilities [2], all of which relate to the skills mentioned above and thus can help cultivate them. Unfortunately, fewer physicians are interested in pursuing academic medicine careers [3]. This may be due to an absence of guidance and mentorship within the field [4], a lack of understanding of academic medicine career paths and how to pursue them [5], and more. In response to this general decline, different institutions have created various programs and workshops for faculty members [6], residents [7], and medical students [8] to promote exposure and interest in academic medicine. Some schools, such as the Vanderbilt University School of Medicine, have created one-year programs focusing on basic science and wet laboratory research to pique student interest in becoming a physician-scientist [9]. Other schools like McGill University [10], the University of California San Francisco [11], and the University of Michigan Medical School [12] have successfully integrated a program for faculty and students to pursue development specifically in medical education. Some researchers have also hosted workshops at various conferences to expose the participants to academic medicine [3, 5, 13]. Regardless of how an institution decides to implement its program or workshop, it has been evident that at least the introduction to academic medicine increases interest and promotes consideration in becoming a part of this field [13]. Participants in these kinds of programs and workshops have also been shown to respond positively to their efficacy and outcomes [14], as shown through the results of post-program/workshop questionnaires or self-administered surveys [15]. The need to combat the ongoing shortage of physicians in academic medicine prevails. Researchers have found that there is a decline on a national scale in academic clinicians despite implementing these institutional pathways at different medical schools [16]. These authors suggest that more focus on support and guidance, earlier exposure to medical students, and maximizing recruitment in these programs may contribute as a more effective method [16]. As such, California University of Science and Medicine (CUSM) has developed its own longitudinal Academic Medicine Scholars Program to provide its participants with early guidance and mentorship to pursue careers in the field. The Scholars Program led the introduction to academic medicine with workshops for both first- and second-year medical student classes in efforts to expose the field early on in their medical careers. To our knowledge, there has not yet been a workshop that focuses on introducing academic medicine to primarily first- and second-year students, especially outside of a conference setting. We sought to progress the work previously done by Fernandez CR et al. [13]. We hypothesize that introducing academic medicine to medical students early in their careers in the medical school setting, beyond conferences, will promote even greater interest in the field.

## Materials and methods

The workshop was organized and led by three medical student co-leads of the Academic Medicine Scholars Program at CUSM. The workshop and the Scholars Program were overseen by two faculty members. We utilized the workshop material from Fernandez CR et al. and edited it to reflect CUSM-specific examples and student interest [13]. The final content of the workshop was approved by the two academic medicine faculty of the Scholars Program. This study was approved by the CUSM Institutional Review Board (approval number: HS-2022-22), and informed consent was obtained from each student participant. The study population consisted of 104 first- and second-year medical students at CUSM. We chose first- and second-year medical students specifically since they can be considered relatively early in their medical careers. Each workshop was conducted for approximately 90 minutes during the students' weekly learning sessions. One workshop was given for the first-year medical students, and another was given separately for the second-year medical students. The workshop consisted of three different components: (1) an informational didactic session using a Microsoft PowerPoint (PPT) presentation about different academic medicine roles and responsibilities, (2) small-group breakout discussion sessions to review case scenarios, and (3) a faculty panel to provide personal anecdotes and advice to students.

Presentation

The PPT presentation consisted of 37 slides and included explanations of different clinical and administrative roles in academic medicine. Aspects such as advantages, challenges, focuses, and expectations were delineated for each role. The PPT also recommended steps to take for academic promotion and allowed students to reflect on how their interests and values may influence their career choice.

Discussion of case scenarios

The students were then divided into groups of five to 10 participants to discuss which of two presented case scenarios of possible summer experiences would better prepare them for a career in academic medicine or if they equally prepared the student. The first case scenario was about a CUSM student interested in pursuing a research project with one of the anatomy professors. The second case scenario was about the same student creating a public health infographic for patients at the school's free clinic. Students were also asked to identify skills they could obtain through both experiences that can help them in the field of academic medicine. The students then provided reasoning for their choice with the rest of the workshop participants.

Faculty panel

Finally, students listened to a panel of CUSM faculty ranging from assistant professors to deans and chairs of medical education as they shared their experiences in the field. The panel moderator provided planned questions. Participants were then allowed to ask their own questions.

Survey 

To assess the response to the workshop session, the students were administered two online surveys, one prior to (pre-) and one after completing the workshop (post-). Each survey took about five minutes to complete. Both surveys consisted of the same five questions, each using a 5-point Likert scale (1=strongly disagree to 5=strongly agree). The first question asked the participants to indicate how much confidence they have in their ability to start building a foundation for a career in academic medicine. The next question asked the participants to indicate how much confidence they have in their ability to identify an academic medicine career goal that aligns with their professional interests. The students were then asked to indicate how much confidence they have in their ability to explain to a peer the field of academic medicine. Question four then asked the students how much interest they have in pursuing a career in academic medicine. The last question asked the students how much they would like to learn more about the field of academic medicine.

After recording their answers to the above questions, participants were asked two open-ended follow-up questions regarding the workshop in the post-workshop survey. The first question asked participants what they liked about this academic medicine workshop. The second question asked participants what they would have liked to see and would have done differently in the academic medicine workshop.

Data analysis

We analyzed data using IBM SPSS Statistics 27.0 for Windows (IBM Corp., Armonk, New York, US). We compared pre- and post-survey question means with each other using a paired t-test. We also compared survey responses within both classes and between first- and second-year students.

## Results

Eighty-three (80%) first-year medical students and 21 (20%) second-year medical students attending CUSM participated in their respective workshops for a total of 104 pre-clerkship students. A total of 83 (80%) first-year students responded to the pre-workshop survey, and 76 (73%) first-year students responded to the post-workshop survey. A total of 21 (20%) second-year students responded to the pre-workshop survey, and 20 (19%) second-year students responded to the post-workshop survey. Questions one through four had an increase in the survey response means for the post-workshop survey compared to the pre-workshop survey. We found there was a statistical significance in pre- and post-survey responses for these questions (p < 0.001, p < 0.001, p < 0.001, p < 0.001); however, question five pre- and post-survey responses were not statistically significant (p = 0.78), as shown in Table 1.

**Table 1:** Mean difference in survey scores among all student participants at CUSM (N=104). CUSM: California University of Science and Medicine.

	Mean Pre-Survey Score^a^ (SD)	Mean Post-Survey Score^a^ (SD)	Mean Difference (95% CI)	P-value
Question 1	2.6 (1.1)	3.5 (1.0)	0.9 (0.6-1.2)	<0.001
Question 2	2.8 (1.1)	3.6 (1.1)	0.8 (0.5-1.1)	<0.001
Question 3	2.5 (1.2)	3.8 (0.9)	1.2 (0.9-1.5)	<0.001
Question 4	2.8 (1.1)	3.4 (1.1)	0.6 (0.3-0.9)	<0.001
Question 5	3.6 (1.1)	3.6 (1.1)	0.0 (-0.3-0.3)	0.78
^a^Rated on a 5-point Likert Scale (1=Strongly Disagree, 5=Strongly Agree)				

Fifty-nine (57%) students responded to the first short-answer question at the end of the post-survey response, "Please share what you liked about this academic medicine workshop." Almost all the responses to this question were positive, with a majority highlighting the academic medicine faculty panel. One student wrote, "I really enjoyed hearing about the different perspectives on how all the different faculty got to where they are." Another said, "Loved the panel!! So interesting and helpful to hear differing pathways, and comforting to see how they've all gotten to where they want to in different ways and without always knowing what they wanted at the start." Regarding the presentation given, workshop participants claimed, "I liked how the students explained the different roles of academic medicine and what they entailed because I feel like many medical students hear about these positions but don't necessarily know exactly what they are. I also appreciated the Q&A," and "Clearly explained and compared the similarities and differences between different paths, which improved my understanding drastically." Twenty-three (22%) students responded with constructive feedback to the second short-answer question at the end of the post-survey response, "Please share what you would have liked to see and would have done differently in this academic medicine workshop." Some responses included, "I would like to see more business admin discussion whether that be for the school or potentially hospital." To have a more holistic faculty panel, students suggested having "someone from the C-Suite role." Many students wrote that the case scenario discussion was of "limited value" and "less necessary," wishing that they could have spent more time asking the "faculty members questions." Moreover, some participants "would have liked to get a little bit of information on the scholars program itself instead of spending time on a group activity."

## Discussion

According to the responses to the post-workshop short-answer questions, the academic medicine faculty panel was the main contributing factor in promoting interest. Generally, utilizing personal or first-hand experiences easily piques interest [17], potentially leading to further engagement with the field. This could especially be true if the individuals sharing their experiences are already perceived as role models or mentors to the learners [18]. Therefore, we suggest that future workshops introducing academic medicine should focus on including those in the field who can explain how and why they got to their position. Our two workshops included three-to-five professionals with titles ranging from Assistant Professor to the Chair of Medical Education to provide a variety of perspectives. While the participants appreciated the panel's diversity, some suggested including individuals with administrative and C-Suite roles. The feedback from participants suggested that some preferred more time for the faculty panel and less or no time for small-group scenario discussions. Although the case scenarios were altered from the original source material to be more engaging and relatable, learners may have been more interested in hearing about personal experiences to understand the field of academic medicine better. Real-world examples and advice from faculty who have successfully navigated the field can be more impactful for learners than hypothetical discussions with peers. Additionally, as the two scenarios presented in the workshop were equally effective in demonstrating critical skills, individual interest plays a crucial role in determining students' paths. Hearing about faculty lives and opinions can be a unique and valuable experience compared to case scenario discussions, which may be more available in other learning opportunities. Many of these kinds of workshops are presented at conferences that have specific populations. The conference attendees may then choose to participate in these workshops. Therefore, the participants at, for example, a research conference may already be interested in academic medicine. We conducted our workshop on campus during a required class for medical students who may or may not have heard about academic medicine. Doing this allowed us to have a broader audience who may not have any knowledge about the field of academic medicine. A broad target audience was crucial since our workshop aimed to increase interest and overall knowledge of the field of academic medicine among future medical professionals. This also allowed us to easily expose many medical students to academic medicine through a short-term method. Our findings above also highlight the necessity of mentors. This long-term method can provide early exposure and guidance to medical students to help increase interest in academic medicine and encourage more medical professions to pursue academic medicine. Some of the participants' feedback suggested more discussion on CUSM's Academic Medicine Scholars Program and about resources to gain more knowledge and experiences in academic medicine. Therefore, workshops should not be the only means to introduce students to academic medicine. CUSM created its Academic Medicine Scholars Program based on the evidence from other institutions that these programs are effective in helping its students and promoting interest in the field [8]. Specifically, our program consists of faculty and student leads who guide the program's participants and provide mentorship. The 1.5-2 year program includes journal clubs on articles pertaining to the field, interactive online modules teaching different aspects of academic medicine, a practicum experience in which students apply the knowledge they have learned through the modules, and a longitudinal capstone project. The faculty oversees the students' capstone projects that are required for the completion of the program. Several factors limit our study. First, there was a significant difference in the number of participants between the first- and second-year classes, which may have impacted the results. This disparity may have been influenced by the timing of the second-year workshop, which occurred right before exams. In the future, presenters may want to consider scheduling workshops at times when students have fewer academic obligations. Additionally, there was a slight decrease in the number of post-workshop survey responses compared to the pre-workshop survey responses. This may have been due to participants leaving the workshop early or not having enough time to complete the survey. To address this issue, future surveys should remain open long after the workshop has concluded to allow for more time for students to provide feedback. Future directions for this work could include assessing the long-term impact of the Academic Medicine Scholars Program on participants' career choices and academic success. Additionally, hosting these kinds of workshops and programs among various medical schools allows for the opportunity to conduct a multicentric study that can further promote our findings. Finally, investigating the potential benefits and drawbacks of incorporating academic medicine education into medical school curricula could provide implications for the training of future physicians.

## Conclusions

Our goal was to introduce academic medicine to students early in their medical careers to foster interest in the field. We believed that a didactic PPT explaining academic medicine and its career roles, case scenario discussions, and a diverse faculty panel would be useful in achieving this goal. Based on the overall increase in survey response means in the post-workshop survey compared to the pre-workshop survey, we determine that the workshops were successful in developing interest and understanding of academic medicine among the pre-clerkship students. In conclusion, workshops could be a valuable introduction to a general group of students that can then be followed by enrollment in academic medicine programs for those interested in further pursuing a career in academia. We suggest workshops targeted to medical students early in their careers to foster interest sooner and to allow more time for opportunities, such as joining specific academic programs.
